# Morphological and multi-gene phylogenetic analyses reveal five new hyphomycetes from freshwater habitats

**DOI:** 10.3389/fmicb.2023.1253239

**Published:** 2023-12-05

**Authors:** Ya-Zhou Zhang, Qi-Lei Chen, Jian Ma, Yong-Zhong Lu, Hu-Biao Chen, Ning-Guo Liu

**Affiliations:** ^1^College of Pharmacy, Guizhou University of Traditional Chinese Medicine, Guiyang, Guizhou, China; ^2^School of Chinese Medicine, Hong Kong Baptist University, Hong Kong, Hong Kong SAR, China; ^3^School of Food and Pharmaceutical Engineering, Guizhou Institute of Technology, Guiyang, Guizhou, China

**Keywords:** 5 new taxa, asexual morph, biodiversity, morphology, phylogeny

## Abstract

During the survey on freshwater hyphomycetes in Guangxi, Guizhou and Hainan Provinces, China, five fresh collections were encountered. Based on their morphology, these five isolates were identified as belonging to *Hermatomyces*, *Kirschsteiniothelia*, *Paramonodictys*, *Pleopunctum* and *Sparticola*. Multi-gene phylogenetic analyses were performed for each genus, which resulted in the identification of five new species, namely *Hermatomyces hainanensis*, *Kirschsteiniothelia ramus*, *Paramonodictys globosa*, *Pleopunctum guizhouense*, and *Sparticola irregularis*. Detailed descriptions and illustrations of the morphological characteristics of these new taxa were provided. This research enriches the biodiversity of freshwater dematiaceous hyphomycetes.

## 1 Introduction

Freshwater fungi are a diverse and heterogeneous group that can be classified into different classes (Marvanová, 1980; Goh and Hyde, 1996; Shearer et al., 2009; Baschien et al., 2013; Luo et al., 2019; Dong et al., 2020; Yang et al., 2023). Calabon et al. (2022) listed 3,870 species occurring in freshwater habitats. They play essential roles, such as decomposers for submerged woody debris, in freshwater ecosystems (Wong et al., 1998; Grossart et al., 2019), and many of them possess unique biochemical properties that have great potential for various applications (Krauss et al., 2011; El-Elimat et al., 2021). Therefore, the study of fungal biodiversity in freshwater habitats is important, and five genera are involved in the present paper.

*Hermatomyces* was introduced by Spegazzini (1910) with the type species *H. tucumanensis*. This genus was previously placed in Lophiotremataceae (Tibpromma et al., 2016; Doilom et al., 2017). Hashimoto et al. (2017) excluded *Hermatomyces* from Lophiotremataceae and resurrected the family Hermatomycetaceae based on their phylogenetic analyses using SSU, ITS, LSU, *tef1-α* and *rpb2* sequences. Asexual morph of *Hermatomyces* species is characterized by sporodochial conidiomata and dimorphic conidia, i.e., cylindrical conidia and lenticular conidia (Ellis, 1971; Chang, 1995; Tibpromma et al., 2016; Doilom et al., 2017; Koukol et al., 2018; Ren et al., 2021). Its sexual morph was recently reported by de Silva et al. (2022), which has dark brown to black ascomata with central ostiole, 8-spored, bitunicate asci, and broadly fusiform, hyaline, 1-septate ascospores.

Hawksworth (1985) proposed *Kirschsteiniothelia* as a sexual morphic genus, which was linked to the *Dendryphiopsis* asexual morph. Kirschsteiniotheliaceae was established by Boonmee et al. (2012) after observing the sexual-asexual morph connection based on a culture study. The order Kirschsteiniotheliales was subsequently proposed by Hernández-Restrepo et al. (2017). The dendryphiopsis-like asexual morph is characterized by macronematous, mononematous, apically branched conidiophores and cylindrical conidia with rounded ends (Ellis, 1971; Pratibha et al., 2010; Boonmee et al., 2012). Su et al. (2016) identified *Kirschsteiniothelia submersa* as a sporidesmium-like asexual morph, and then several novel *Kirschsteiniothelia* species with sporidesmium-like morphology were introduced, such as *K. aquatica*, *K. cangshanensis*, *K*. *fluminicola* and *K. rostrata* (Hyde et al., 2017; Bao et al., 2018). Sun et al. (2021) provided a detailed summary of *Kirschsteiniothelia* species.

Hyde et al. (2020) introduced *Paramonodictys* for a monodictys-like species, which is characterized by present stroma and globose to subglobose, brown, muriform conidia. *Paramonodictys* is classified in Parabambusicolaceae (Pleosporales) (Hyde et al., 2020). To date, four species are accepted in this genus (Hyde et al., 2020; Yang et al., 2022; Xu et al., 2023), among which, *P. dispersa*, has been reported from a freshwater habitat (Xu et al., 2023).

*Pleopunctum* belonging to Phaeoseptaceae (Pleosporales) was introduced by Liu et al. (2019) based on the type species *Pl*. *ellipsoideum*, along with *Pl*. *pseudoellipsoideum*. Seven species were included in *Pleopunctum*, of which three species, *viz. Pl*. *megalosporum*, *Pl*. *multicellularum* and *Pl*. *rotundatum* were reported from freshwater habitats (Xu et al., 2023). The sexual morph of *Pleopunctum* has not been reported, and its asexual morph is characterized by sporodochial conidiomata, oval to ellipsoidal, brown, muriform conidia often with one or several hyaline, globose to ellipsoidal basal cells (Liu et al., 2019; Phukhamsakda et al., 2020; Boonmee et al., 2021; Senwanna et al., 2021; Wanasinghe et al., 2022; Xu et al., 2023). Hyaline phragmosporous or dictyosporous conidia have also been reported in this genus (Phukhamsakda et al., 2020; Senwanna et al., 2021; Wanasinghe et al., 2022).

*Sparticola* was introduced by Phukhamsakda et al. (2016) to accommodate *S. forlicesenae*, *S. junci* (type species) and *S. triseptata*. A fourth species, *S. muriformis*, was introduced by Karunarathna et al. (2017). All four of these terrestrial species have been found to exhibit sexual morphs in nature. Only *S. junci* produces a hyphomycetous asexual morph in culture, characterized by semi-macronematous to macronematous, pale brown to brown conidiophores, holoblastic conidiogenous cells, and irregular, brown to dark brown conidia (Phukhamsakda et al., 2016).

In this study, we introduce five new species collected from freshwater habitats in Guangxi, Guizhou and Hainan Provinces, China. Based on morphological characteristics and phylogenetic analyses, they are identified as *Hermatomyces hainanensis* sp. nov., *Kirschsteiniothelia ramus* sp. nov., *Paramonodictys globosa* sp. nov., *Pleopunctum guizhouense* sp. nov., and *Sparticola irregularis* sp. nov. Detailed descriptions and illustrations are provided for these five new taxa.

## 2 Materials and methods

### 2.1 Collections and examination of specimens

Fresh samples were collected from May 2021 to July 2022 in Guangxi, Guizhou and Hainan Provinces, China. The samples were incubated in moist plastic boxes at room temperature for 14 days. A Motic SMZ 168 Series dissecting microscope was used to check the specimen. Fruiting bodies of the new collections were examined and photographed with a Nikon ECLIPSE Ni compound microscope fitted with a Canon 90D digital camera. The software Tarosoft (R) Image Frame Work was used to take measurements of fungal structures, and Adobe Photoshop CC 2019 (Adobe Systems, USA) was used to prepare the photo-plates.

Single conidium isolations were carried out on potato dextrose agar (PDA) media (Senanayake et al., 2020). Germinated conidia were individually transferred to fresh PDA media plates and incubated in a constant temperature incubator at 25°C. Dried specimens were deposited in the Herbarium of Cryptogams, Kunming Institute of Botany Academia Sinica (HKAS), Kunming, China, and the herbarium of Guizhou Academic of Agriculture Sciences (GZAAS), Guiyang, China. Pure cultures were deposited in the Guizhou Culture Collection (GZCC), Guiyang, China. Fungal Names numbers were applied in Fungal Names (2023).^1^

### 2.2 DNA extraction, PCR amplification and sequencing

Genomic DNA was extracted from fresh mycelia growing on PDA medium for 1 month at 25°C using a DNA extraction kit (BioFlux, China). Four different gene regions, the nuclear large subunit rDNA (28S, LSU), the internal transcribed spacer (ITS), the translation elongation factor (*tef1-α*), and the RNA polymerase II subunit 2 (*rpb2*) were selected for study. Primer pairs LR0R/LR5 (Vilgalys and Hester, 1990), ITS5/ITS4 (White et al., 1990), EF1-983F/EF1-2218R (Rehner and Buckley, 2005) and fRPB2-5F/fRPB2-7cR (Liu et al., 1999) were used to amplify part of LSU, ITS, *tef1-α* and *rpb2* loci, respectively. Polymerase chain reaction (PCR) was carried out in a 50 μL reaction volume containing 44 μL of 1.1 × T3 Supper PCR Mix (Qingke Biotech, China), 2 μL of forward and reverse primers, and 2 μL of DNA template. The PCR protocols were referred to Ma et al. (2023). And 1% agarose electrophoresis gels stained with ethidium bromide were used to examine the resulting PCR products. Successful PCR products were sequenced by Beijing Qingke Biotechnology Co., Ltd.

### 2.3 Phylogenetic analyses

Sequences obtained from different primers were analyzed with related taxa determined by blastn search in NCBI. Alignments for different gene loci were automatically performed by online MAFFT version 7.^2^ Trimal v1.2 (Capella-Gutierrez et al., 2009) was used to remove ambiguously aligned regions and uninformative positions with gappyout option. Multi-gene alignments were combined using SequenceMatrix 1.7.8 (Vaidya et al., 2011). Alignments were checked visually using AliView (Larsson, 2014). Sequences derived in this study were deposited in GenBank (Table 1).

**Table 1:** Taxa used in this study and GenBank accession numbers.

Species	Strain number	LSU	ITS	*tef1-α*	*rpb*2	*tub*2
*Angustimassarina populi*	MFLUCC 13–0034	KP888642	KP899137	KR075164	NA	N/A
*Anteaglonium globosum*	ANM 925.2	GQ221879	N/A	GQ221925	N/A	N/A
*Anteaglonium parvulum*	MFLUCC 14–0821	KU922915	N/A	KU922921	N/A	N/A
*Aquastroma magniostiolatum*	CBS 139680	NG_056936	LC014540	AB808486	N/A	N/A
*Corynespora cassiicola*	CBS 100822	GU301808	N/A	GU349052	N/A	N/A
*Corynespora torulosa*	CPC 15989	NG_058866	NR_145181	N/A	N/A	N/A
*Decaisnella formosa*	BCC 25617	GQ925847	N/A	GU479850	N/A	N/A
*Decaisnella formosa*	BCC 25616	GQ925846	N/A	GU479851	N/A	N/A
*Exosporium stylobatum*	CBS 160.30	JQ044447	JQ044428	N/A	N/A	N/A
*Forliomyces uniseptata*	MFLUCC 15–0765	KU721762	KU721772	N/A	N/A	N/A
*Hermatomyces amphisporus*	CBS 146610	LR812664	LR812664	N/A	N/A	N/A
*Hermatomyces amphisporus*	CBS 146613	LR812662	LR812662	LR812657	LR812668	LR812673
*Hermatomyces amphisporus*	CBS 146614	LR812666	LR812666	LR812660	LR812671	LR812676
*Hermatomyces anomianthi*	MFLUCC 21–0202	OK655817	OL413437	OM117546	N/A	N/A
*Hermatomyces bauhiniae*	MFLUCC 16–0395	MK443378	MK443382	MK443384	MK443386	N/A
*Hermatomyces biconisporus*	KUMCC 17–0183	MH260296	MH275063	MH412771	MH412755	N/A
*Hermatomyces bifurcatus*	CCF 5899	LS398262	LS398262	LS398416	LS398343	LS398441
*Hermatomyces bifurcatus*	CCF 5900	LS398263	LS398263	LS398417	LS398344	LS398442
*Hermatomyces clematidis*	MFLUCC 17–2085	MT214556	MT310603	MT394735	MT394684	N/A
*Hermatomyces constrictus*	CCF 5904	LS398264	LS398264	LS398418	LS398345	LS398443
*Hermatomyces indicus*	MFLUCC 14–1143	KU764692	KU144920	KU872754	KU712488	N/A
*Hermatomyces indicus*	MFLUCC 14–1144	KU764693	KU144921	KU872755	KU712489	N/A
*Hermatomyces iriomotensis*	KH 361	LC194367	LC194483	LC194394	LC194449	N/A
*Hermatomyces jinghaensis*	HKAS 112167	MW989519	MW989495	MZ042642	N/A	N/A
*Hermatomyces krabiensis*	MFLUCC 16–0249	KX525742	KX525750	KX525758	KX525754	N/A
*Hermatomyces krabiensis*	MFLUCC 16–2817	KY559394	N/A	N/A	N/A	N/A
***Hermatomyces hainanensis***	**GZCC 23–0592**	**OR091329**	**OR098708**	N/A	N/A	N/A
*Hermatomyces megasporus*	CCF 5897	N/A	LS398265	LS398419	LS398346	LS398444
*Hermatomyces megasporus*	CCF 5898	LS398266	LS398266	LS398420	N/A	LS398445
*Hermatomyces nabanheensis*	KUMCC 16–0149	KY766059	KY766058	KY766061	N/A	N/A
*Hermatomyces pandanicola*	MFLUCC 16–0251	KX525743	KX525751	KX525759	KX525755	N/A
*Hermatomyces reticulatus*	CCF 5893	LS398267	LS398267	LS398421	LS398347	LS398446
*Hermatomyces reticulatus*	MFLUCC 15–0843	KX259523	KX259521	KX259527	KX259529	N/A
*Hermatomyces sphaericoides*	CCF 5908	LS398273	LS398273	LS398427	LS398352	LS398450
*Hermatomyces sphaericoides*	CCF 5895	LS398270	LS398270	LS398424	LS398350	LS398447
*Hermatomyces sphaericus*	PMA 116080	LS398281	LS398281	LS398431	LS398356	LS398454
*Hermatomyces sphaericus*	PMA 116081	N/A	LS398283	LS398432	LS398357	LS398455
*Hermatomyces sphaericus*	PRC 4105	N/A	LS398286	N/A	N/A	N/A
*Hermatomyces sphaericus*	PRC 4104	N/A	LS398278	LS398430	LS398355	LS398453
*Hermatomyces sphaericus*	KZP 462	N/A	LS398287	LS398434	LS398359	LS398457
*Hermatomyces sphaericus*	MFLUCC 16–2818	KY559393	N/A	N/A	N/A	N/A
*Hermatomyces sphaericus*	MFLUCC 16–0266	KX525740	KX525748	KX525756	KX525752	N/A
*Hermatomyces sphaericus*	MFLUCC 14–1140	KU764695	KU144917	KU872757	KU712486	N/A
*Hermatomyces trangensis*	BCC 80741	KY790600	KY790598	KY790606	KY790604	N/A
*Hermatomyces trangensis*	BCC 80742	KY790601	KY790599	KY790607	KY790605	N/A
*Hermatomyces tucumanensis*	CCF 5912	LS398288	LS398288	LS398435	LS398360	LS398458
*Hermatomyces tucumanensis*	CCF 5915	LS398290	LS398290	LS398437	LS398362	N/A
*Hermatomyces turbinatus*	MFLUCC 21–0038	MW989518	MW989494	MZ042641	MZ042638	MZ042645
*Hermatomyces verrucosus*	CCF 5903	LS398292	LS398292	LS398439	LS398364	LS398462
*Hermatomyces verrucosus*	CCF 5892	LS398291	LS398291	LS398438	LS398363	LS398461
*Kirschsteiniothelia acutispora*	MFLU 21–0127	ON980758	OP120780	N/A	N/A	N/A
*Kirschsteiniothelia aethiops*	CBS 109.53	AY016361	N/A	N/A	N/A	N/A
*Kirschsteiniothelia aethiops*	MFLUCC 16–1104	MH182589	MH182583	N/A	N/A	N/A
*Kirschsteiniothelia aethiops*	S-783	MH182595	MH182586	N/A	N/A	N/A
*Kirschsteiniothelia aethiops*	MFLUCC 15–0424	KU500578	KU500571	N/A	N/A	N/A
*Kirschsteiniothelia aquatica*	MFLUCC 17–1685	MH182594	MH182587	N/A	N/A	N/A
*Kirschsteiniothelia arasbaranica*	IRAN 2509C	KX621987	KX621986	N/A	N/A	N/A
*Kirschsteiniothelia arasbaranica*	IRAN 2508C	KX621984	KX621983	N/A	N/A	N/A
*Kirschsteiniothelia cangshanensis*	MFLUCC 16–1350	MH182592	MH182584	N/A	N/A	N/A
*Kirschsteiniothelia crustacea*	MFLU 21–0129	MW851854	MW851849	N/A	N/A	N/A
*Kirschsteiniothelia dushanensis*	GZCC 19–0415	N/A	OP377845	N/A	N/A	N/A
*Kirschsteiniothelia extensa*	MFLU 21–0126	ON980757	OP120779	N/A	N/A	N/A
*Kirschsteiniothelia fluminicola*	MFLUCC 16–1,263	MH182588	MH182582	N/A	N/A	N/A
*Kirschsteiniothelia lignicola*	MFLUCC 10–0036	HQ441568	HQ441567	N/A	N/A	N/A
*Kirschsteiniothelia nabanheensis*	HJAUP C2004	OQ023273	OQ023197	N/A	N/A	N/A
*Kirschsteiniothelia nabanheensis*	HJAUP C2006	OQ023275	OQ023274	N/A	N/A	N/A
*Kirschsteiniothelia phoenicis*	MFLUCC 18–0216	MG860484	MG859978	N/A	N/A	N/A
***Kirschsteiniothelia ramus***	**GZCC 23–0596**	**OR091333**	**OR098711**	**OR494046**	**OR494049**	N/A
*Kirschsteiniothelia rostrata*	MFLUCC 15–0619	KY697276	KY697280	N/A	N/A	N/A
*Kirschsteiniothelia rostrata*	MFLUCC 16–1124	MH182590	N/A	N/A	N/A	N/A
*Kirschsteiniothelia septemseptata*	MFLU 21–0126	ON980757	OP120779	N/A	N/A	N/A
*Kirschsteiniothelia spatiosa*	MFLU 21–0128	N/A	OP077294	N/A	N/A	N/A
*Kirschsteiniothelia submersa*	MFLUCC 15–0427	KU500577	KU500570	N/A	N/A	N/A
*Kirschsteiniothelia submersa*	S-481	MH182591	N/A	N/A	N/A	N/A
*Kirschsteiniothelia submersa*	S-601	MH182593	MH182585	N/A	N/A	NA
*Kirschsteiniothelia tectonae*	MFLUCC 12–0050	KU764707	KU144916	N/A	N/A	N/A
*Kirschsteiniothelia thailandica*	MFLUCC 20–0116	MT984443	MT985633	N/A	N/A	N/A
*Kirschsteiniothelia thujina*	JF 13210	KM982718	KM982716	N/A	N/A	N/A
*Kirschsteiniothelia xishuangbannaensis*	ZHKUCC 22–0220	OP303181	OP289566	N/A	N/A	N/A
*Kirschsteiniothelia xishuangbannaensis*	ZHKUCC 22–0221	OP303182	OP289563	N/A	N/A	N/A
*Lignosphaeria fusispora*	MFLUCC 11–0377	KP888646	KP899140	N/A	N/A	N/A
*Lignosphaeria thailandica*	MFLUCC 11–0376	KP888645	KP899139	N/A	N/A	N/A
*Lonicericola fuyuanensis*	MFLU 19–2,850	NG_073809	NR_172419	MN938324	N/A	N/A
*Lonicericola hyaloseptispora*	KUMCC 18–0149	NG_066434	NR_164294	N/A	N/A	N/A
*Lonicericola qujingensis*	GMB 1386	NG_154015	NR_182717	OM857556	N/A	N/A
*Multilocularia bambusae*	MFLUCC 11–0180	NG_059654	NR_148099	KU705656	N/A	N/A
*Multiseptospora thailandica*	MFLUCC 11–0183	NG_059554	NR_148080	KU705657	N/A	N/A
*Multiseptospora thysanolaenae*	MFLUCC 11–0202	NG_059655	NA	KU705658	N/A	N/A
*Neoaquastroma bauhiniae*	MFLUCC 16–0398	NG_067814	NR_165217	MH028247	N/A	N/A
*Neoaquastroma guttulatum*	MFLUCC 14–0917	KX949740	KX949739	KX949742	N/A	N/A
*Parabambusicola aquatica*	MFLUCC 18–1140	NG_073791	NR_171877	N/A	N/A	N/A
*Parabambusicola bambusina*	MAFF 239462	AB807536	LC014578	AB808511	N/A	N/A
*Parabambusicola thysanolaenae*	KUMCC 18–0147	NG_066435	NR_164044	MK098209	N/A	N/A
*Paradictyoarthrinium diffractum*	MFLUCC 12–0557	KP744497	KP744454	N/A	N/A	N/A
*Paradictyoarthrinium tectonicola*	MFLUCC 12–0556	KP744499	KP744456	N/A	N/A	N/A
*Paramonodictys dispersa*	KUNCC 10788	OQ146988	ON261165	OQ943185	N/A	N/A
*Paramonodictys dispersa*	KUNCC 10782	OQ146982	ON261159	OQ943183	N/A	N/A
*Paramonodictys dispersa*	KUNCC 10783	OQ146983	ON261160	OQ943184	N/A	N/A
***Paramonodictys globosa***	**GZCC 23–0594**	**OR091331**	N/A	**OR494045**	**OR494048**	N/A
*Paramonodictys hongheensis*	KUMCC 21–0343	NG_081549	ON350762	OL505582	N/A	N/A
*Paramonodictys hongheensis*	KUMCC 21–0346	OL436224	OL436235	OL505583	N/A	N/A
*Paramonodictys solitarius*	GZCC 20–0007	MN897835	MN901152	MT023012	N/A	N/A
*Paramonodictys yunnanensis*	KUMCC 21–0337	OL436226	OL436231	OL505585	N/A	N/A
*Paramonodictys yunnanensis*	KUMCC 21–0347	OL436228	OL436233	OL505586	N/A	N/A
*Paratrimmatostroma kunmingense*	KUN HKAS 102224	MK098196	MK098192	MK098208	N/A	N/A
*Phaeoseptum aquaticum*	CBS 123113	JN644072	N/A	N/A	N/A	N/A
*Phaeoseptum terricola*	MFLUCC 10–0102	MH105779	N/A	MH105781	N/A	N/A
*Phyllobathelium anomalum*	MPN 242	GU327722	N/A	N/A	N/A	N/A
*Phyllobathelium firmum*	ERP 3175	GU327723	N/A	N/A	N/A	N/A
*Pleopunctum bauhiniae*	MFLUCC 17–2091	NG_073849	NR_170810	MT394632	N/A	N/A
*Pleopunctum ellipsoideum*	MFLU 19–0685	MK804517	MK804512	MK828510	N/A	N/A
***Pleopunctum guizhouense***	**GZCC 23–0595**	**OR091332**	**OR098710**	N/A	N/A	N/A
*Pleopunctum heveae*	MFLUCC 21-0146a	OL782070	OL780491	N/A	N/A	N/A
*Pleopunctum heveae*	MFLUCC 21-0146b	OL782071	OL780492	N/A	N/A	N/A
*Pleopunctum megalosporum*	KUNCC 10785	OQ146985	ON261162	OQ943186	N/A	N/A
*Pleopunctum megalosporum*	KUNCC 10442	OQ146986	OQ135180	OQ943187	N/A	N/A
*Pleopunctum menglaense*	KUMCC 21–0025	ON009102	ON009118	ON009261	N/A	N/A
*Pleopunctum menglaense*	KUMCC 21–0026	ON009103	ON009119	ON009262	N/A	N/A
*Pleopunctum multicellularum*	KUNCC 10789	OQ146989	ON261166	OQ943190	N/A	N/A
*Pleopunctum multicellularum*	KUNCC 10781	OQ146981	ON261158	OQ943189	N/A	N/A
*Pleopunctum multicellularum*	KUNCC 10778	OQ146978	ON261155	N/A	N/A	N/A
*Pleopunctum pseudoellipsoideum*	MFLU 19–0686	MK804518	MK804513	MK828511	N/A	N/A
*Pleopunctum pseudoellipsoideum*	KUMCC 21–0820	ON009100	ON009116	ON009259	N/A	N/A
*Pleopunctum pseudoellipsoideum*	HKAS122915	ON009101	ON009117	ON009260	N/A	N/A
*Pleopunctum rotundatum*	KUNCC 10787	OQ146987	ON261164	OQ943194	N/A	N/A
*Pleopunctum rotundatum*	KUNCC 10780	OQ146980	ON261157	OQ943193	N/A	N/A
*Pleopunctum thailandicum*	MFLUCC 21–0039	MZ198896	MZ198894	MZ172461	N/A	N/A
*Preussia flanaganii*	CBS 112.73	NG_064098	NR_077168	N/A	N/A	N/A
*Preussia funiculata*	CBS 659.74	GU301864	N/A	N/A	N/A	N/A
*Preussia lignicola*	CBS 363.69	DQ384098	GQ203783	N/A	N/A	N/A
*Preussia lignicola*	CBS 264.69	GU301872	N/A	N/A	N/A	N/A
*Preussia minima*	CBS 524.50	MH868263	MH856741	N/A	N/A	N/A
*Preussia* sp.	ELV3.11	KF269205	JN418774	N/A	N/A	N/A
*Preussia* sp.	ELV3.2	KF269206	JN418773	N/A	N/A	N/A
*Pseudomonodictys aquatica*	MFLUCC 22–0018	ON553406	ON561291	ON556673	N/A	N/A
*Pseudomonodictys tectonae*	MFLUCC 12–0552	NG_059590	N/A	KT285571	N/A	N/A
*Sparticola forlicesenae*	MFLUCC 14–1097	KU721763	KU721773	N/A	N/A	N/A
*Sparticola forlicesenae*	MFLUCC 14–0952	KU721764	KU721774	N/A	N/A	N/A
*Sparticola junci*	MFLUCC 15–0030	KU721765	KU721775	N/A	N/A	N/A
*Sparticola junci*	MFLUCC 13–0926	KU721766	KU721776	N/A	N/A	N/A
*Sparticola muriformis*	MFLUCC 17–0316	KY768862	KY768864	N/A	N/A	N/A
*Sparticola triseptata*	CBS 614.86	EF165031	N/A	N/A	N/A	N/A
*Sporormia fimetaria*	UPS:Lundqvist 2302c	GQ203728	GQ203768	N/A	N/A	N/A
*Sporormia fimetaria*	UPS:dissing Gr.81.194	GQ203729	GQ203769	N/A	N/A	N/A
***Sparticola irregularis***	**GZCC 23–0593**	**OR091330**	**OR098709**	**OR494044**	**OR494047**	N/A
*Sporormurispora atraphaxidis*	MFLUCC 17–0742	MG829083	MG828971	N/A	N/A	N/A
*Sporormurispora pruni*	MFLUCC 17–0803	MG829084	MG828972	N/A	N/A	N/A
*Thyridaria macrostomoides*	GKM1033	GU385190	N/A	GU327776	N/A	N/A
*Thyridaria macrostomoides*	GKM1159	GU385185	N/A	GU327778	N/A	N/A
*Thyridaria macrostomoides*	GKM224N	GU385191	N/A	GU327777	N/A	N/A
*Trichophoma cylindrospora*	CBS 146340	LR732024	LR732023	N/A	N/A	N/A
*Westerdykella angulata*	CBS 610.74	NG_057754	NR_155956	N/A	N/A	N/A
*Westerdykella dispersa*	CBS 297.56	NG_057827	NR_111187	N/A	N/A	N/A
*Westerdykella ornata*	CBS 379.55	MH869059	MH857522	N/A	N/A	N/A
*Xenomonodictys iranica*	CBS 147181	MW175406	MW175368	N/A	N/A	N/A

Newly generated sequences are indicated in bold. *N/A, data not available in GenBank.

Maximum likelihood (ML) analyses were performed using IQ-TREE web server (Trifinopoulos et al., 2016). Substitution model was automatically tested. Ultrafast bootstrap (BS) analysis was implemented with 1,000 replicates. Maximum likelihood bootstrap values (ML-BS) equal or greater than 75% are marked near each node.

Bayesian inference (BI) analyses were carried out in MrBayes 3.2.6 (Ronquist et al., 2012) using a Markov Chain Monte Carlo (MCMC) algorithm. The best-fit substitution model GRT + I + G was decided for all four gene regions by MrModeltest 2.3 (Nylander, 2008) under the Akaike Information Criterion (AIC). Two parallel runs of four simultaneous Markov chains were performed for 1,000,000 generations. Trees were sampled every 1,000th generations. Burn-in phase was set at 25% and the remaining trees were used for calculating posterior probabilities (PP). PP values equal or greater than 0.95 are marked near each node.

Trees were visualized with FigTree v1.4.4,^3^ and the layouts were edited using Adobe Illustrator CS6 software (Adobe Systems, USA).

## 3 Taxonomy

**Figure 1:**
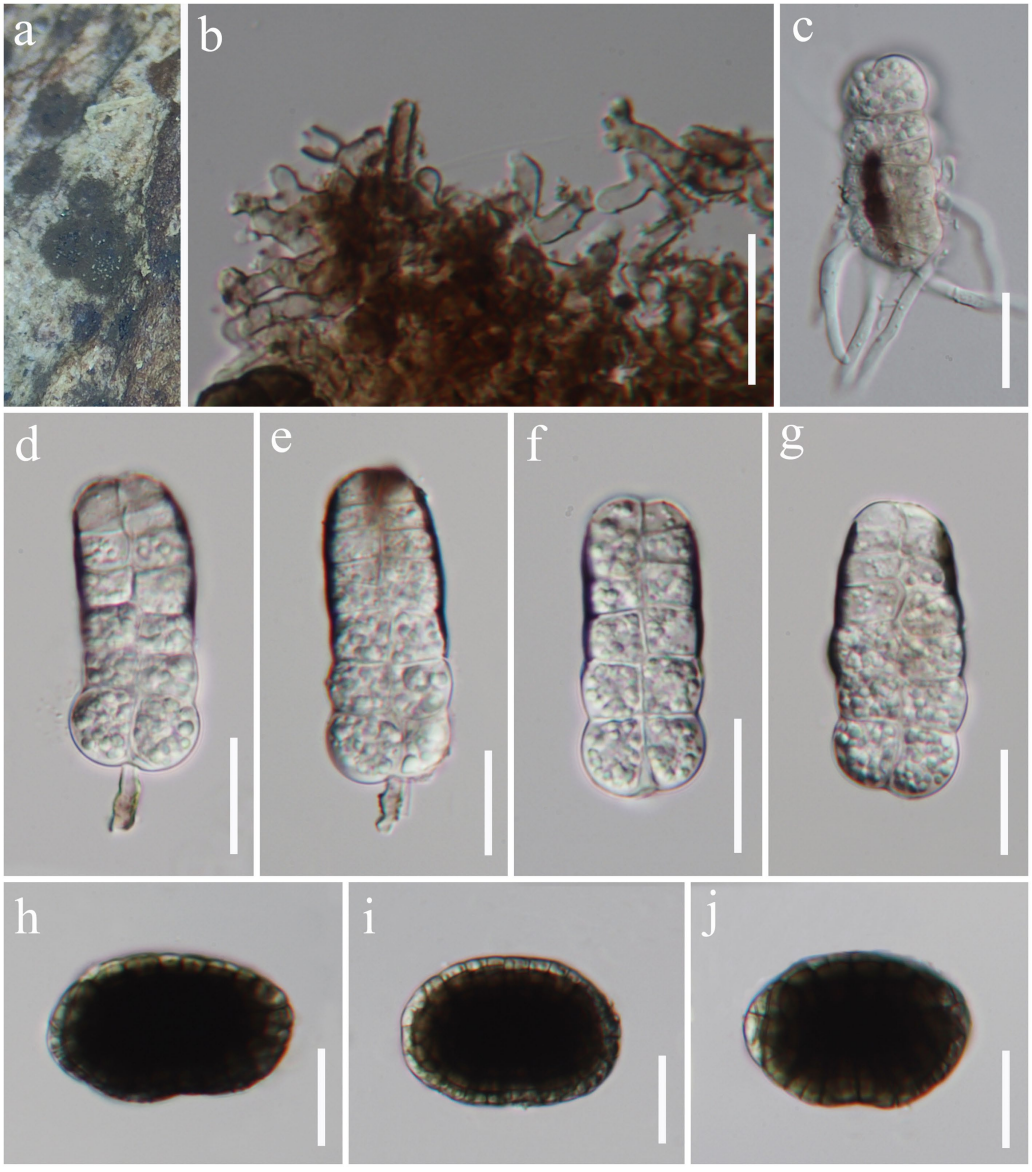
*Hermatomyces hainanensis* (HKAS 129170, holotype). **(A)** Colonies on natural substrates. **(B)** Subicular hyphae. **(C)** Germinated conidium. **(D–G)** Cylindrical conidia. **(H–J)** Lenticular conidia. Scale bars: **(B–J)** = 20 μm.

*Hermatomyces hainanensis* J. Ma, Y.Z. Zhang & Y.Z. Lu, sp. nov., Figure 1.

Fungal Names number: FN571666.

Holotype: HKAS 129170.

Etymology: Referring to the location where the species was collected.

*Saprobic* on decaying wood in freshwater habitat. Sexual morph: Undetermined. Asexual morph: hyphomycetous. *Colonies* on natural substrate sporodochial, effuse, scattered, circular or subcircular, consisting of a brown sterile mycelial outer zone and an abundantly sporulating, dark brown to blackish brown center. *Mycelium* partly immersed, partly superficial, composed of pale to brown, branched, septate hyphae. *Conidiophores* 15–19 × 2–5 μm ( $\bar{x}$ = 48 × 32 μm, *n* = 15), micronematous to semi-macronematous, unevenly cylindrical, geniculate, subhyaline to brown, septate, thick-walled. *Conidiogenous cells* 4.5–8 × 2.5–3 μm ( $\bar{x}$ = 6.5 × 2.5 μm, *n* = 15), monoblastic, integrated, terminal, subcylindrical, subhyaline to pale brown. *Conidia* dimorphic, (1) cylindrical conidia 51–67 × 16–24 μm ( $\bar{x}$ = 58.5 × 21 μm, *n* = 30), hyaline to subhyaline, often with a distinct dark brown pigmentation from the top downwards or at rim of the conidia, straight or broadly curved, phragmoseptate or muriform, sometimes with oblique septa, constricted at the septa, consisting of two columns from one or two basal cells, rounded at the apex; (2) lenticular conidia 44–52 × 29–39 μm ( $\bar{x}$ = 48 × 32 μm, *n* = 30), ellipsoidal in front view, central cells dark brown to blackish brown, peripheral cells subhyaline to pale olivaceous brown, forming a distinct ring, muriform, constricted at the septa, smooth-walled, side views not observed.

Culture characteristics: Conidia were germinated on PDA medium and produced germ tubes within 18 h. Colonies grown on PDA are pale brown to brown, circular, surface flat, edge entire, reaching 36 mm diam. in 42 days at 25°C.

Material examined: China, Hainan Province, Qiongzhong Li and Miao Autonomous County, Baihualing Rainforest cultural tourism area, 18°98′ N, 109°82′ E, on rotting wood in a freshwater stream, 29 December 2021, Jian Ma, BH39 (HKAS 129170, holotype; GZAAS 23–0595, isotype), ex-type living culture GZCC 23-0592.

**Figure 2:**
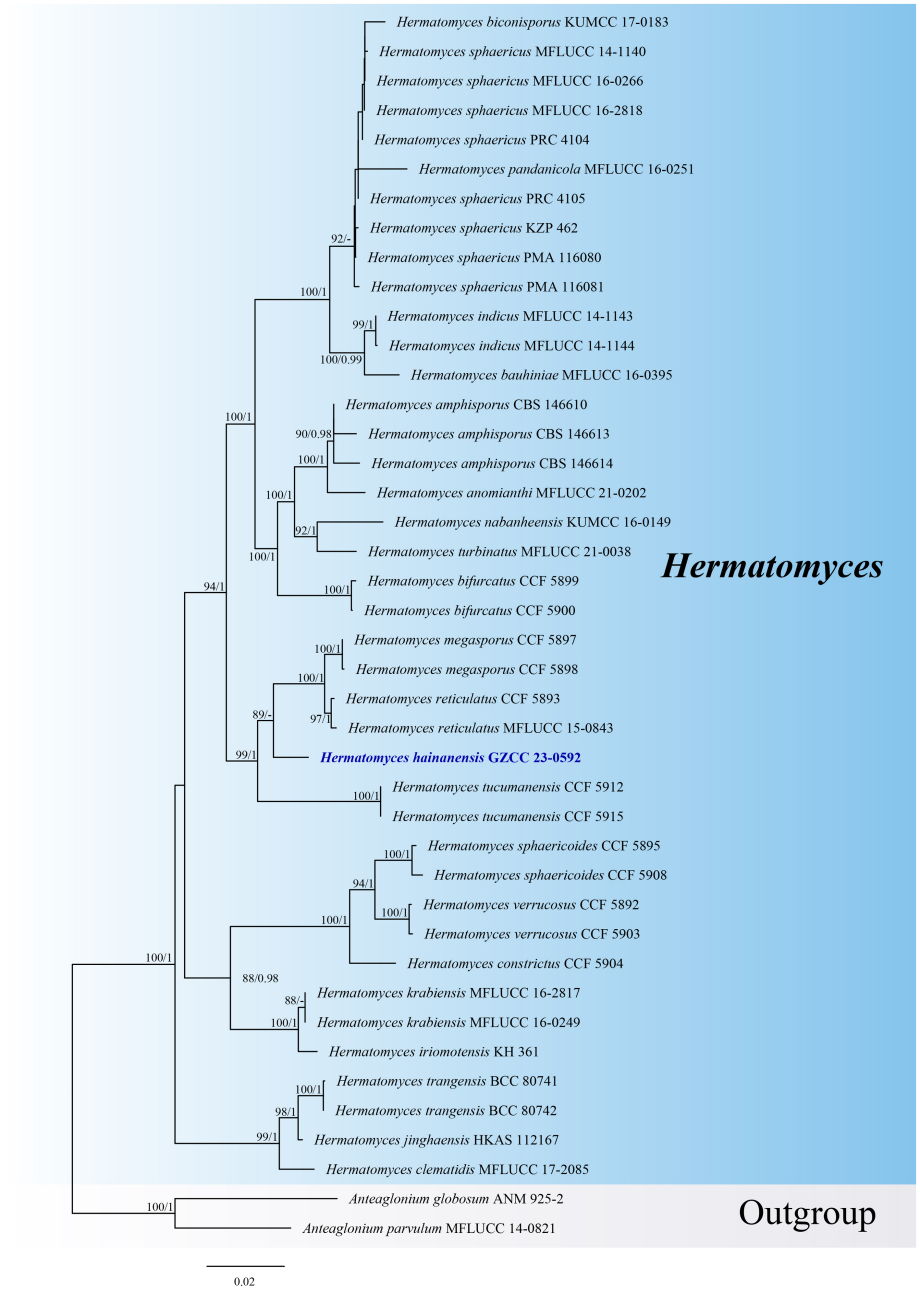
ML tree (−ln = 12797.020) based on the combined LSU-ITS-*tef1-α*-*rpb2*-*tub* rDNA sequences. The combined dataset comprises 41 strains, including the new collection. The alignment comprises 3,940 characters (LSU: 1–852, ITS: 853–1324, *tef1-α*: 1325–2260, *rpb2*: 2261–3295, tub: 3296–3940) including gaps. Among them, number of constant sites are 3,150, and number of parsimony informative sites are 597. Bootstrap support values for ML greater than 75% and PP greater than 0.95 are given near nodes as ML-BS/PP. The tree is rooted with *Anteaglonium globosum* (ANM 925–2) and *Anteaglonium parvulum* (MFLUCC 14–0821). The new taxon is indicated in bold and blue.

Notes: *Hermatomyces hainanensis* is similar to other *Hermatomyces* species with dimorphic conidia, such as *H. bifurcatus*, *H. constrictus*, *H. iriomotensis*, *H. jinghaensis*, *H. krabiensis*, *H. megaspores*, *H. tucumanensis*, and *H. turbinatus* (Chang, 1995; Tibpromma et al., 2016, 2017; Hashimoto et al., 2017; Koukol et al., 2018; Ren et al., 2021). Based on phylogenetic analyses, *Hermatomyces hainanensis* (GZCC 23-0592) is closely related to *H. megasporus* (CCF 5897 and CCF 5898) and *H. reticulatus* (CCF 5893 and MFLUCC 15-0843), although *H. reticulatus* only exhibits one type of conidia (Koukol et al., 2018). Despite some overlap in the sizes of the cylindrical (51–67 × 16–24 μm vs. 49.5–60.5 × 18–28 μm) and lenticular (44–52 × 29–39 μm vs. 49–56 × 37–46 μm) conidia of *Hermatomyces hainanensis* and *H. megasporus* (Koukol et al., 2018), the phylogenetic analyses suggest that they are distinct species (Figure 2). Comparisons of ITS sequences showed that there are 22 bp (in a total 886 bp, 2.5%) differences with 2 gaps between *H. hainanensis* (GZCC 23-0592) and *H. megasporus* (CCF 5898), and 10 bp (in a total 444 bp, 2.3%) differences with 1 gap between *H. reticulatus* (CCF 5893). Following the guidelines for species delineation (Jeewon and Hyde, 2016), we identify our collection as a new species.

**Figure 3:**
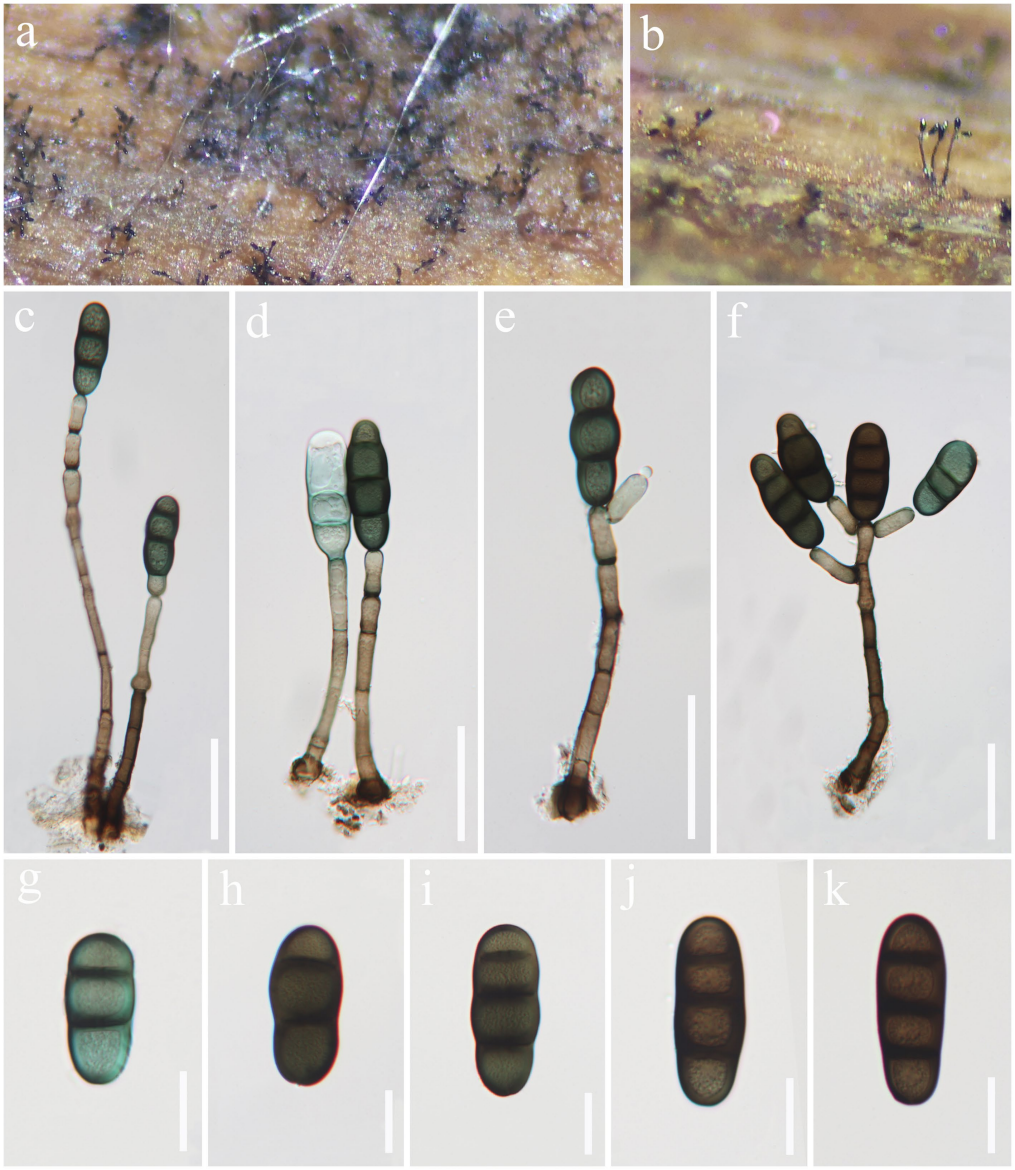
*Kirschsteiniothelia ramus* (HKAS 129167, holotype). **(A,B)** Colonies on natural substrates. **(C–F)** Conidiophores and conidia. **(G–K)** Conidia. Scale bars: **(C–F)** = 50 μm, **(G–K)** = 20 μm.

*Kirschsteiniothelia ramus* J. Ma, Y.Z. Zhang & Y.Z. Lu, sp. nov., Figure 3.

Fungal Names number: FN571667.

Holotype: HKAS 129167.

Etymology: Referring to the apically branched conidiophores.

*Saprobic* on decaying wood in freshwater habitat. Sexual morph: Undetermined. Asexual morph: hyphomycetous. *Colonies* on natural substrate effuse, dark brown, gregarious, velvety. *Mycelium* mostly immersed, composed of gray to brown, branched, septate hyphae. *Conidiophores* 102–248 × 5–11 μm ( $\bar{x}$ = 174 × 8.5 μm, *n* = 15), macronematous, mononematous, erect, straight or flexuous, cylindrical, brown, slightly paler toward the apex, simple or mostly apically branched, septate, thick-walled. *Conidiogenous cells* 18–27 × 6.5–9 μm ( $\bar{x}$ = 22.5 × 8 μm, *n* = 30), monotretic, integrated, terminal at the apex of stipe and fertile branches, pale brown to brown, doliiform or lageniform. *Conidia* 42–56 × 15–22 μm ( $\bar{x}$ = 49.5 × 19.5 μm, *n* = 30), acrogenous, solitary, cylindrical, rounded at the apex, subtruncate at the base, pale olivaceous when young, brown when mature, 2–3-septate, with septa thickened and darkened, verruculose.

Culture characteristics: Conidia were germinated on PDA medium and produced germ tubes within 24 h. Colonies grown on PDA are gray to olivaceous, circular, surface flat, edge entire, reaching 20 mm diam. in 28 days at 25°C.

Material examined: China, Hainan Province, Yanoda Tropical rainforest scenic area, on submerged decaying wood in a freshwater stream, 23 October 2021, Jian Ma, Y13 (HKAS 129167, holotype; GZAAS 23-0599, isotype), ex-type living culture, GZCC 23-0596.

**Figure 4:**
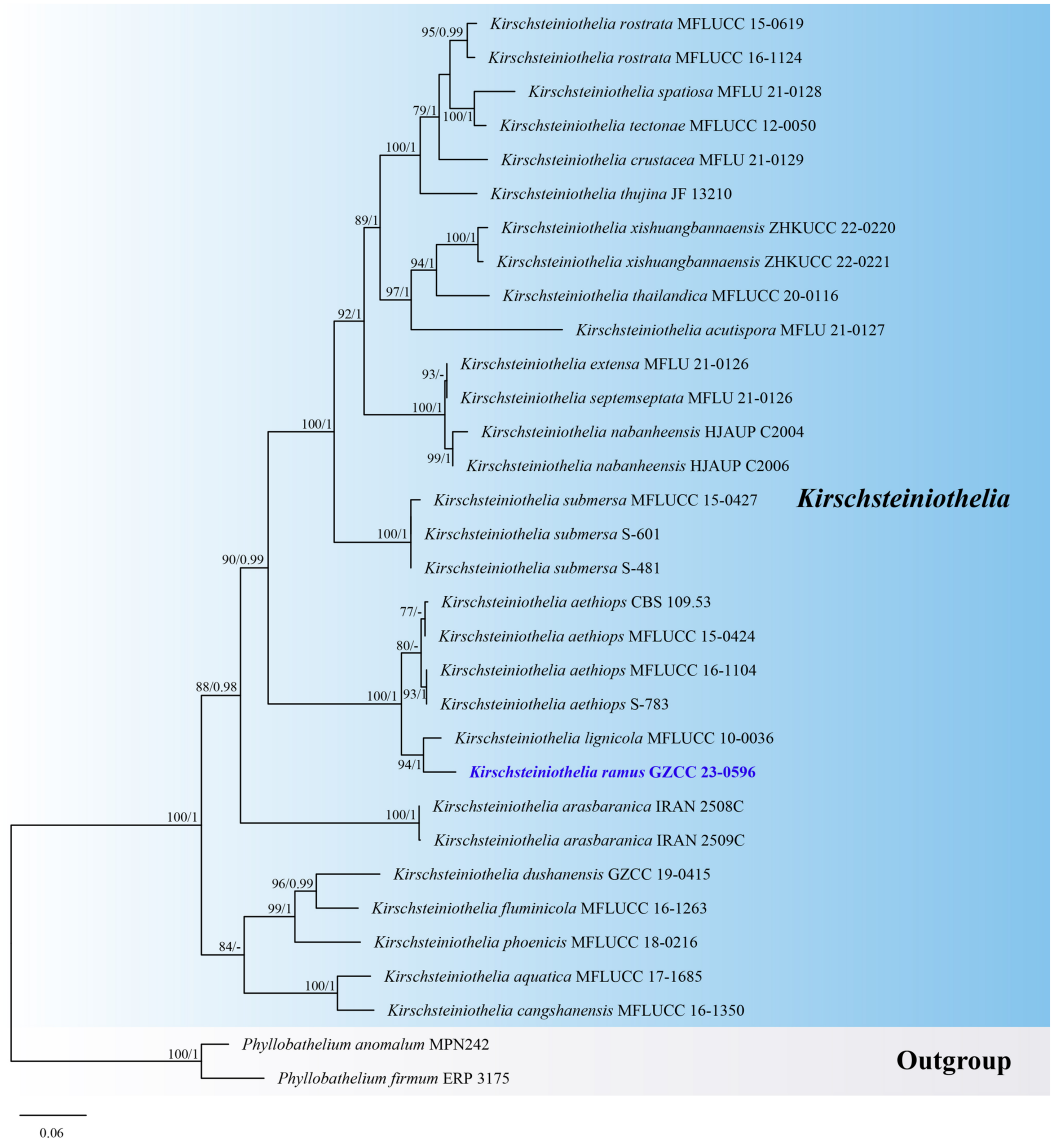
ML tree (−ln = 9010.980915) based on the combined LSU-ITS rDNA sequences. The combined dataset comprises 32 strains, including the new collection. The alignment comprises 1,461 characters (LSU: 1–872, ITS: 873–1461) including gaps. Among them, number of constant sites are 849, and number of parsimony informative sites are 474. Bootstrap support values for ML greater than 75% and PP greater than 0.95 are given near nodes as ML-BS/PP. The tree is rooted with *Phyllobathelium anomalum* (MPN242) and *Phyllobathelium firmum* (ERP 3175). The new taxon is indicated in bold and blue.

Notes: *Kirschsteiniothelia ramus* resembles other *Kirschsteiniothelia* species with dendryphiopsis-like asexual morphs (Sun et al., 2021). Phylogenetically (Figure 4), *Kirschsteiniothelia ramus* (GZCC 23-0596) is a sister taxon to *K. lignicola* (MFLUCC 10-0036). However, *K. ramus* has larger conidiophores (102–248 × 5–11 μm vs. 39–148 × 4–7 μm) and larger conidia (42–56 × 15–22 μm vs. 24.5–35 × 14–16 μm) than *K. lignicola* (Boonmee et al., 2012). Besides, conidia of *K. ramus* are 2–3-septate, while the latter is 1–2-septate (Boonmee et al., 2012). Furthermore, the ITS (471 bp) sequence variation between *K. ramus* (GZCC 23-0596) and *K. lignicola* (MFLUCC 10–0036) occurs in 31 positions, including 5 gaps. Therefore, we introduce *Kirschsteiniothelia ramus* as a new species.

**Figure 5:**
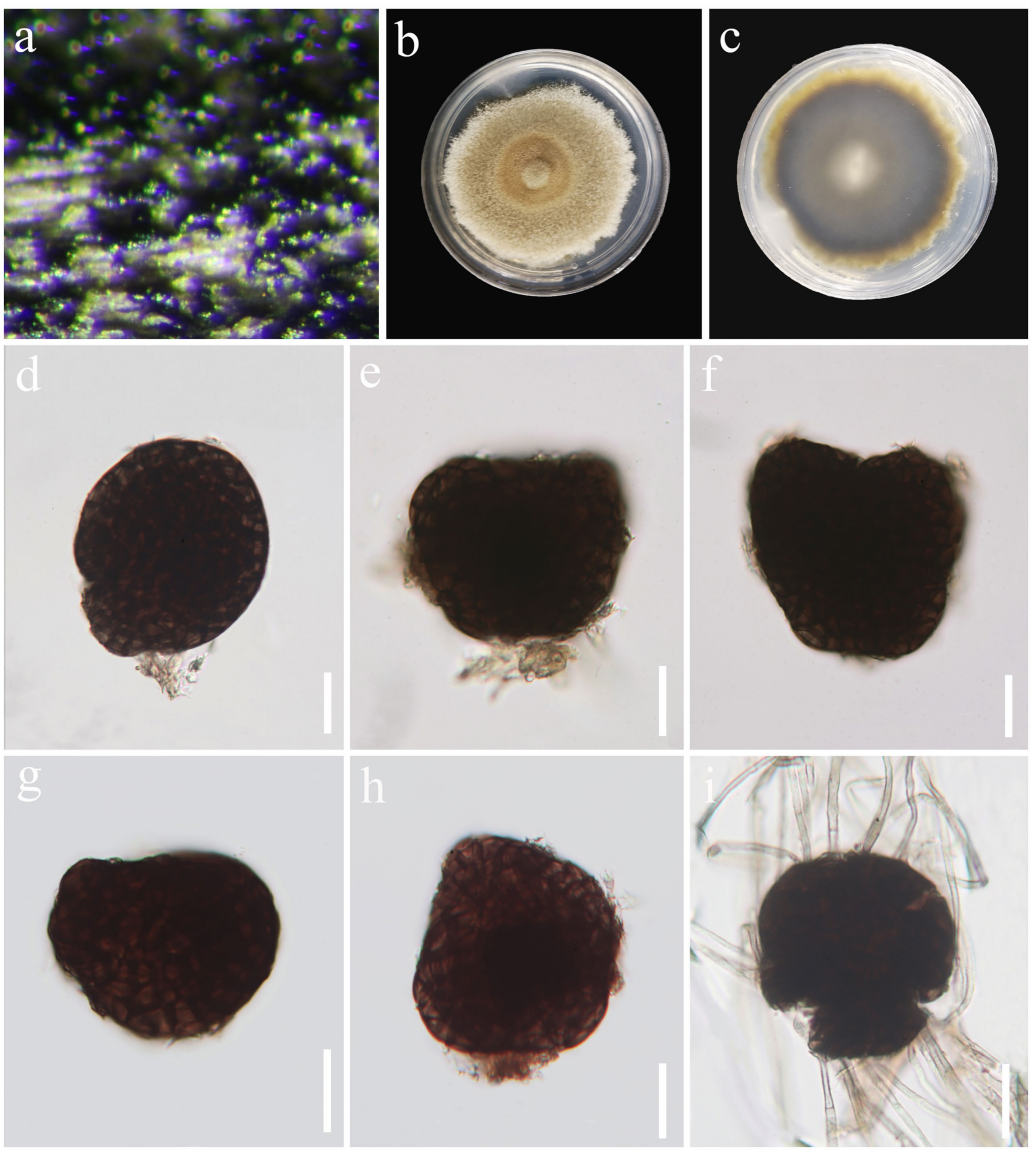
*Paramonodictys globosa* (HKAS 129169, holotype). **(A)** Colonies on natural substrates. **(B,C)** Colonies on PDA media. **(D–H)** Conidia. **(I)** Germinated conidium. Scale bars: **(D–I)** = 20 μm.

*Paramonodictys globosa* J. Ma, Y.Z. Zhang & Y.Z. Lu, sp. nov., Figure 5.

Fungal Names number: FN571668.

Holotype: HKAS 129169.

Etymology: Referring to the globose conidia.

*Saprobic* on decaying wood in freshwater habitat. Sexual morph: Undetermined. Asexual morph: hyphomycetous. *Colonies* on natural substrate superficial, effuse, scattered, black. *Mycelium* mostly immersed, composed of pale brown to brown, branched, septate hyphae. *Stroma* not observed. *Conidiophores* absent. *Conidiogenous cells* monoblastic. *Conidia* 34–65 × 24–60 μm ( $\bar{x}$ = 54 × 46 μm, *n* = 30), solitary, globose to subglobose or irregular, usually broadly rounded at apex, subtruncate at base, olivaceous brown to dark brown, muriform, thickened and darkened at the septa, verrucous.

Culture characteristics: Conidia were germinated on PDA media and produced germ tubes within 24 h. Colonies grown on PDA are pale brown to brown, circular, surface umbonate, edge undulate, reaching 55 mm diam. in 42 days at 25°C.

Material examined: China, Guangxi Zhuang Autonomous Region, Hechi City, Nandan County, Pingzhou, on submerged decaying wood in a freshwater stream, 1 May 2021, Jian Ma, ND22 (HKAS 129169, holotype; GZAAS 23-0597, isotype), ex-type living culture, GZCC 23-0594.

**Figure 6:**
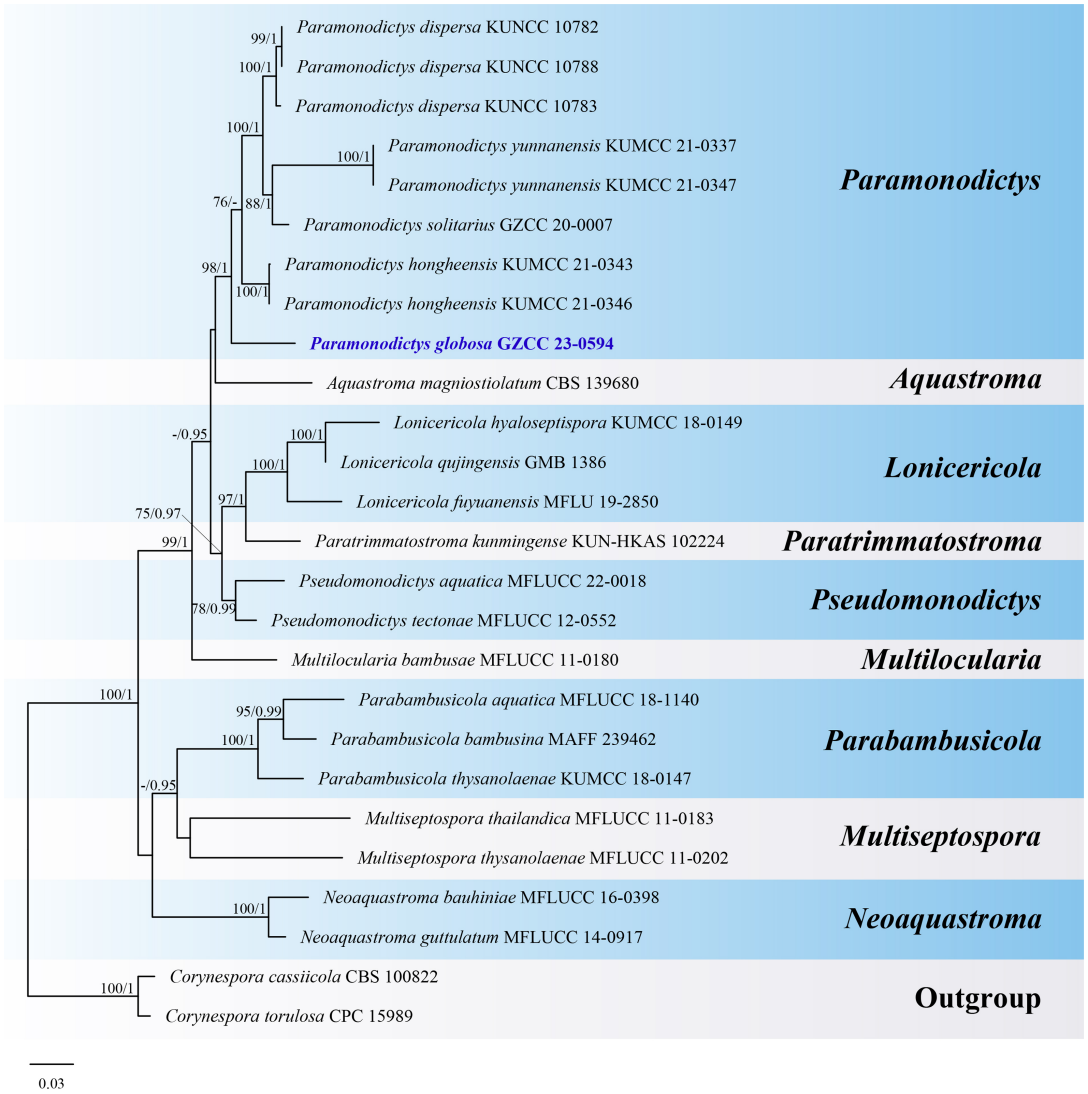
ML tree (−ln = 11817.931619) based on the combined LSU-ITS-*tef1-α* rDNA sequences. The combined dataset comprises 26 strains, including the new collection. The alignment comprises 2,548 characters (LSU: 1–847, ITS: 848–1366, *tef1-α*: 1367–2548) including gaps. Among them, number of constant sites are 1,802, and number of parsimony informative sites are 515. Bootstrap support values for ML greater than 75% and PP greater than 0.95 are given near nodes as ML-BS/PP. The tree is rooted with *Corynespora cassiicola* (CBS 100822) and *Corynespora torulosa* (CPC 15989). The new taxon is indicated in bold and blue.

Notes: A comparison of conidial sizes and shapes for the five accepted *Paramonodictys* species is provided in Table 2. The conidia of *Paramonodictys globosa* are larger than those of *P. hongheensis*. However, *P. globosa* has a similar conidial size to *P. dispersa*, *P. solitarius* and *P. yunnanensis*, meaning they cannot be distinguished based on morphology alone. In our phylogenetic analyses, *P. globosa* (GZCC 23-0594) formed a basal clade within the *Paramonodictys* group (Figure 6), indicating it is phylogenetically distinct. Therefore, we introduce *Paramonodictys globosa* as a new species based on both morphological and molecular evidence.

**Table 2:** Conidial size of accepted *Paramonodictys* species.

Species	Conidial size	Conidial shape	References
*Paramonodictys dispersa*	52–61 × 35–43 μm	Subglobose to elliptical	Xu et al. (2023)
*Paramonodictys globosa*	34–65 × 24–60 μm	Globose to subglobose or irregular	This study
*Paramonodictys hongheensis*	19–26 × 19–22 μm	Subglobose to oval	Yang et al. (2022)
*Paramonodictys solitarius*	50–87 × 40–61 μm	Globose or subglobose	Hyde et al. (2020)
*Paramonodictys yunnanensis*	47–70 × 35–47 μm	Obovoid to subglobose	Yang et al. (2022)

**Figure 7:**
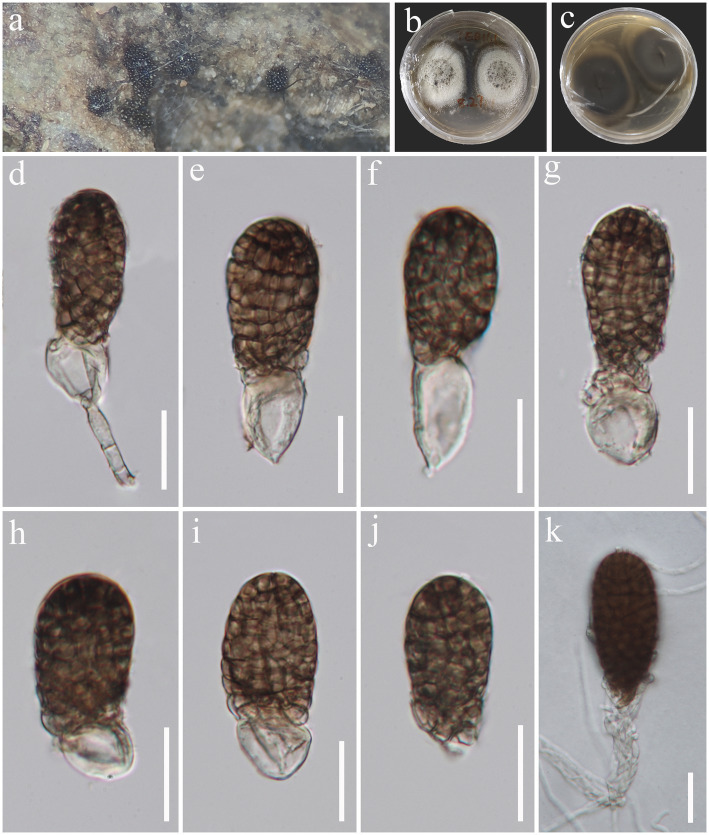
*Pleopunctum guizhouense* (HKAS 129171, holotype). **(A)** Colonies on natural substrates. **(B,C)** Colonies on PDA media. **(D–J)** Conidia. **(K)** Germinated conidium. Scale bars: **(D–I)** = 20 μm.

*Pleopunctum guizhouense* J. Ma, N.G. Liu & Y.Z. Lu, sp. nov., Figure 7.

Fungal Names number: FN571669.

Holotype: HKAS 129171.

Etymology: Referring to the location where the species was collected.

*Saprobic* on decaying wood in freshwater habitat. Sexual morph: Undetermined. Asexual morph: hyphomycetous. *Colonies* on natural substrate superficial, brown, sporodochial, punctiform. *Mycelium* mostly immersed, composed of hyaline to pale brown, branched, septate hyphae. *Conidiophores* macronematous, mononematous, cylindrical, medium brown, simple or branched, septate, thick-walled. *Conidiogenous cells* monoblastic, integrated, terminal, medium brown. *Conidia* 45–64 × 26–29.5 μm ( $\bar{x}$ = 57 × 28 μm, *n* = 30), acrogenous, solitary, oval to ellipsoidal, broadly obtuse at apex, truncate at base, median brown, darker at the apex, muriform, constricted at septa, smooth-walled, often with a hyaline, ellipsoidal to globose basal cell, 16–26 × 11–17 μm ( $\bar{x}$ = 20 × 13.5 μm, *n* = 30).

Culture characteristics: Conidia were germinated on PDA media and produced germ tubes within 12 h. Colonies grown on PDA are white to pale brown in front view and brown in reverse view, circular, surface umbonate, edge undulate, reaching 35 mm diam. in 28 days at 25°C.

Material examined: China, Guizhou Province, Chishui City, Swan Castle Forest Park, on submerged decaying wood in a freshwater stream, 28 July 2022, Jian Ma, TEB11.1 (HKAS 129171, holotype; GZAAS23-0598, isotype), ex-type living culture, GZCC 23-0595.

**Figure 8:**
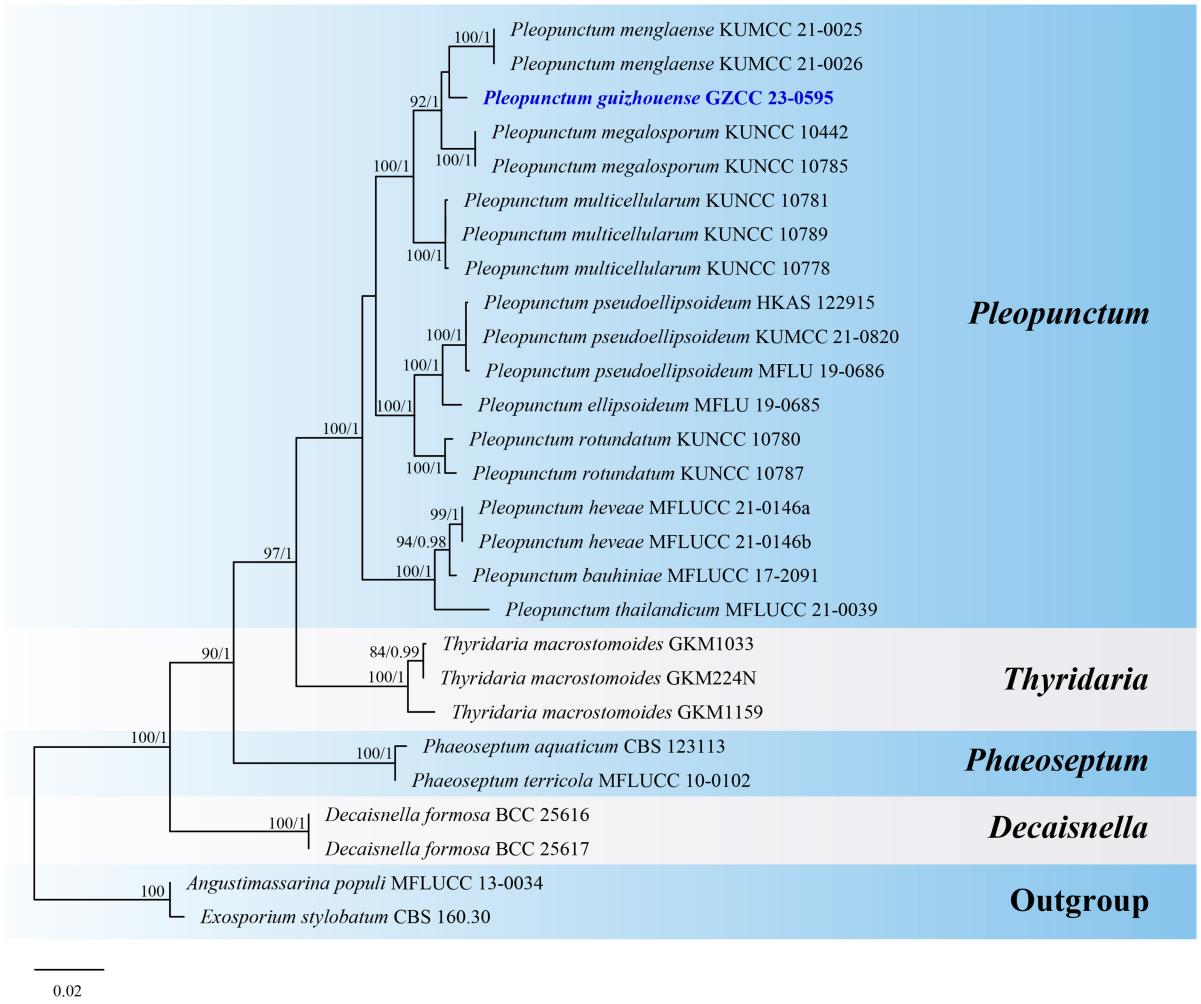
ML tree (−ln = 7049.253556) based on the combined LSU-ITS-*tef1-α* rDNA sequences. The combined dataset comprises 27 strains, including the new collection. The alignment comprises 2,305 characters (LSU: 1–851, ITS: 852–1385, *tef1-α*: 1386–2305) including gaps. Among them, number of constant sites are 1, 822, and number of parsimony informative sites are 411. Bootstrap support values for ML greater than 75% and PP greater than 0.95 are given near nodes as ML-BS/PP. The tree is rooted with *Angustimassarina populi* (MFLUCC 13-0034) and *Exosporium stylobatum* (CBS 160.30). The new taxon is indicated in bold and blue.

Notes: *Pleopunctum guizhouense* (GZCC 23-0595) clusters together with *Pl. menglaense* (KUMCC 21-0025 and KUMCC 21-0026) with a weak support in the phylogenetic analyses (Figure 8). However, it can be differentiated from *Pl. menglaense* by its monomorphic conidia, which are brown and oval to ellipsoidal with a basal cell, while the latter has two types of conidia: spatulate to obovate, hyaline conidia, and brown, ellipsoidal to oblong conidia with 1–3 basal cells (Wanasinghe et al., 2022). The ITS (464 bp) sequence variation between *Pl. guizhouense* (GZCC 23-0595) and *Pl. menglaense* (KUMCC 21-0026) occurs in 16 positions, including 5 gaps. Considering these morphological differences and their distinct phylogenetic positions, we introduce our collection as a new species, named *Pleopunctum guizhouense*.

**Figure 9:**
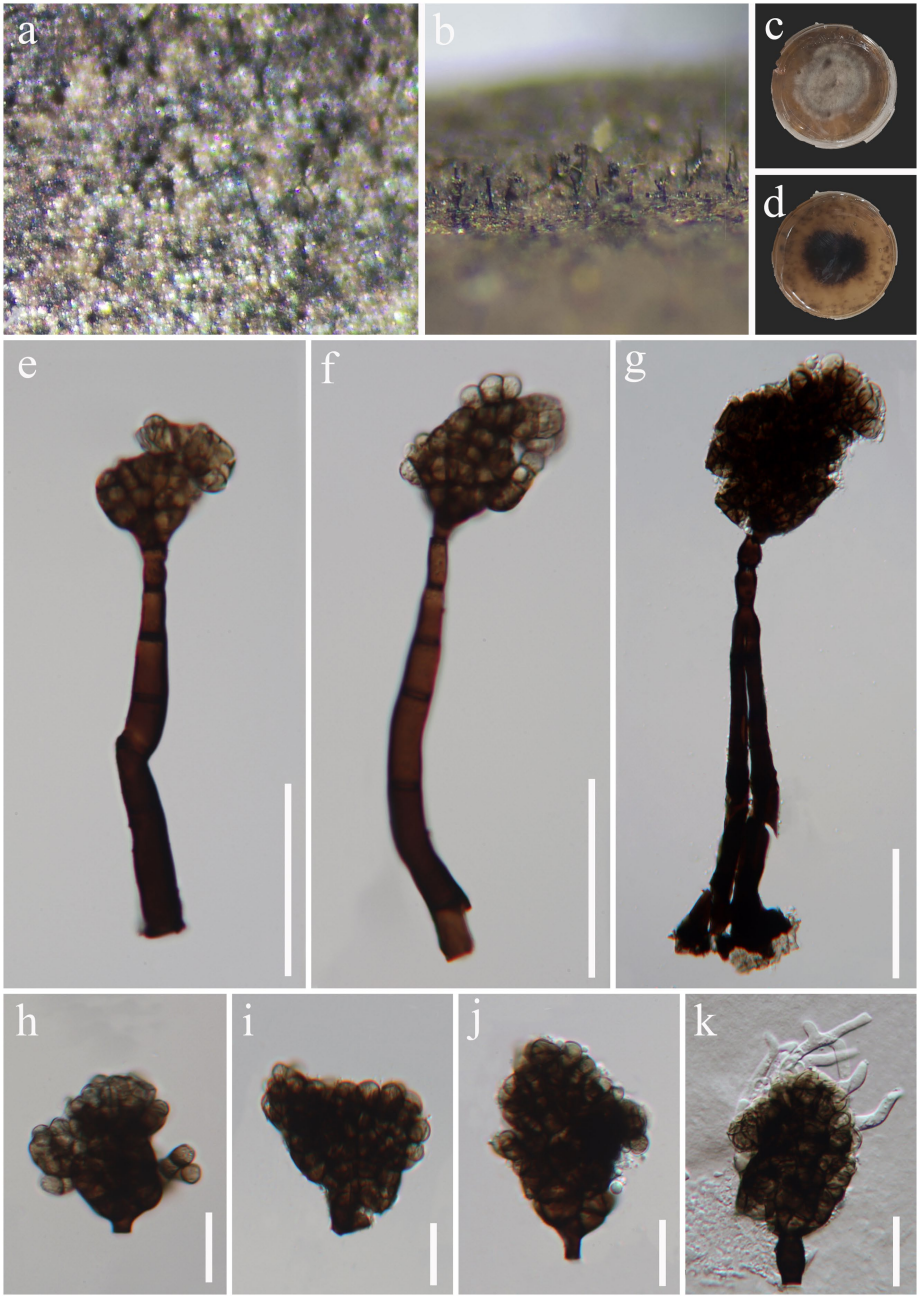
*Sparticola irregularis* (HKAS 129168, holotype). **(A,B)** Colonies on natural substrates. **(C,D)** Colonies on PDA media. **(E–G)** Conidiophores and conidia. **(H–J)** Conidia. **(K)** Germinated conidium. Scale bars: **(E–G)** = 50 μm, **(H–K)** = 20 μm.

*Sparticola irregularis* J. Ma, N.G. Liu & Y.Z. Lu, sp. nov., Figure 9.

Fungal Names number: FN571670.

Holotype: HKAS 129168.

Etymology: Referring to the irregular conidia.

*Saprobic* on decaying wood in freshwater habitat. Sexual morph: Undetermined. Asexual morph: hyphomycetous. *Colonies* on natural substrate effuse, black, velvety. *Mycelium* mostly immersed, composed of pale gray to pale brown, branched, septate hyphae. *Conidiophores* 97–162 × 9–11.5 μm ( $\bar{x}$ = 127 × 10.5 μm, *n* = 15), macronematous, mononematous, erect, subcylindrical, sometimes with doliiform cells at upper part, dark brown and wider at base, paler and thinner toward to the apex, simple, occasionally branched, septate, thick-walled. *Conidiogenous cells* 11–15 × 5–6 μm ( $\bar{x}$ = 13 × 5.5 μm, *n* = 15), monoblastic, integrated, terminal, subcylindrical or doliiform, brown. *Conidia* 33–53 × 33–51 μm ( $\bar{x}$ = 45 × 40 μm, *n* = 30), acrogenous, solitary, irregular, brown to dark brown, muriform, with protuberant, truncate base.

Culture characteristics: Conidia were germinated on PDA media and produced germ tubes within 12 h. Colonies grown on PDA are gray to pale brown, irregular, surface flat, edge filiform, reaching 54 mm diam. in 42 days at 25°C.

Material examined: China, Hainan Province, Haikou City, Xiuying District, Ecological leisure trail, on decaying wood in a freshwater stream, 20°01′ N, 110°25′ E, 10 August 2021, Jian Ma, HK7 (HKAS 129168, holotype; GZAAS23-0596, isotype), ex-type living culture GZCC 23-0593.

**Figure 10:**
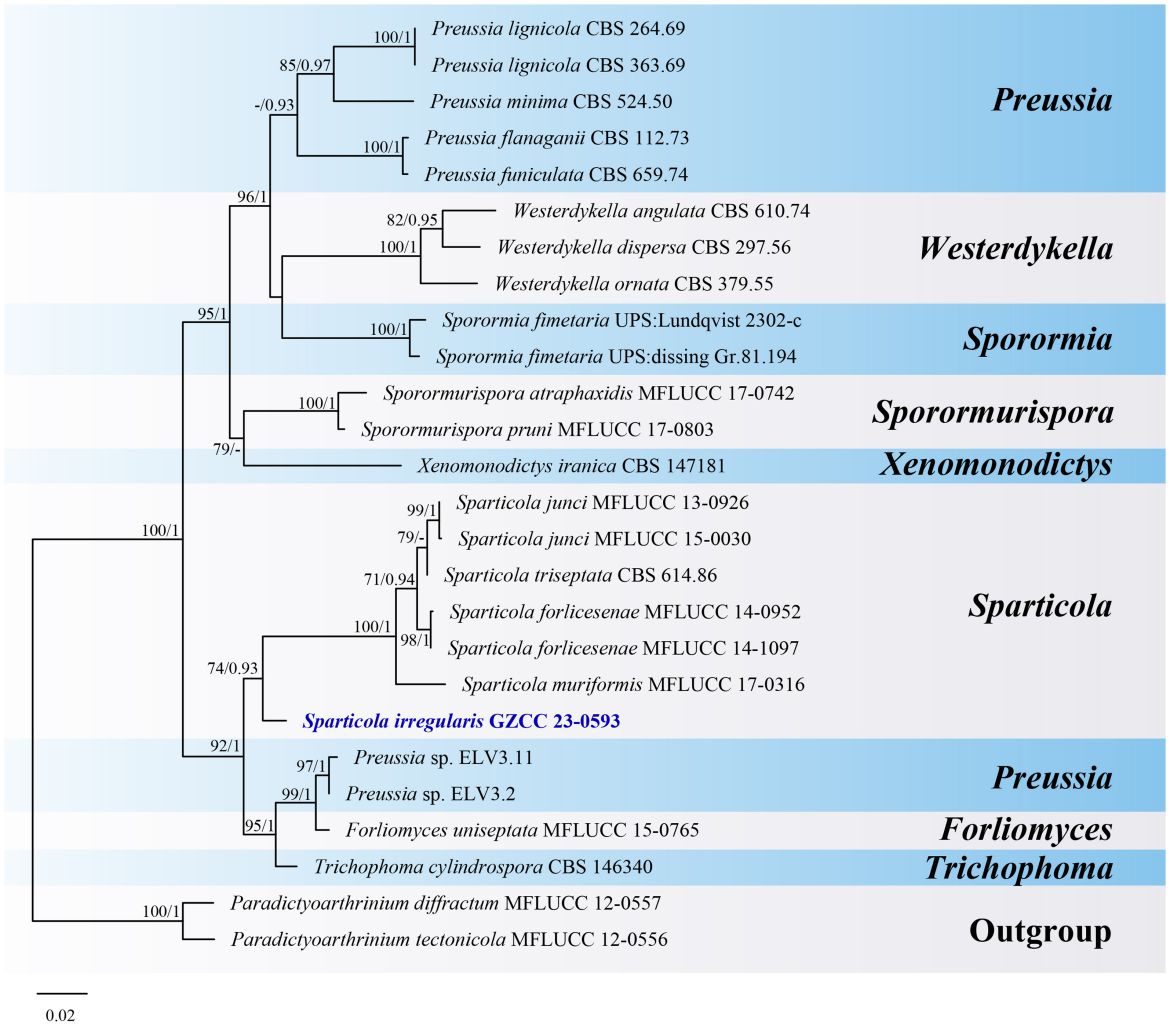
ML tree (−ln = 5776.431900) based on the combined LSU-ITS rDNA sequences. The combined dataset comprises 26 strains, including the new collection. The alignment comprises 1,364 characters (LSU: 1–847, ITS: 848–1364) including gaps. Among them, number of constant sites are 1,039, and number of parsimony informative sites are 265. Bootstrap support values for ML greater than 70% and PP greater than 0.90 are given near nodes as ML-BS/PP. The tree is rooted with *Paradictyoarthrinium diffractum* (MFLUCC 12–0557) and *Paradictyoarthrinium tectonicola* (MFLUCC 12-0556). The new taxon is indicated in bold and blue.

Notes: *Sparticola* species are typically identified by their sexual morphology (Phukhamsakda et al., 2016; Karunarathna et al., 2017). *Sparticola junci* is the only species known to produce a hyphomycetous asexual morphology in culture, which is similar to that of our new collection in terms of conidial morphology (Phukhamsakda et al., 2016). Although our collection has larger conidiophores (97–162 × 9–11.5 μm vs. up to 35 × 4–7 μm) than those of *S. junci*, this difference might be attributed to variations in growth conditions (nature vs. culture). Unfortunately, we were unable to observe sporulation in our culture. Phylogenetically (Figure 10), *Sparticola irregularis* (GZCC 23-0593) forms a basal clade to the *Sparticola* group (ML-BS = 74%, PP = 0.93), and could represent a new genus because the LSU (823 bp) sequences comparison between *S. irregularis* (GZCC 23-0593) and *S. junci* (MFLUCC 15-0030) shows there are 28 position differences, including 2 gaps. However, considering that we only have one isolate, the evidence is insufficient to propose a new genus. Thus, we provisionally assign our new collection to *Sparticola* and introduce the new species *Sparticola irregularis*. Further fresh collections of *Sparticola* species or the discovery of the sexual morphology of *S. irregularis* may provide better resolution for its taxonomic identification.

## 4 Discussion

In this study, we introduce five new species, namely *Hermatomyces hainanensis*, *Kirschsteiniothelia ramus*, *Paramonodictys globosa*, *Pleopunctum guizhouense*, and *Sparticola irregularis*. The discovery of these five new taxa enriches the freshwater fungi resources of China and further reveals the diverse morphology for this group of fungi. *Hermatomyces*, *Kirschsteiniothelia*, *Paramonodictys* and *Pleopunctum* have been reported from both terrestrial and freshwater habitats (Sun et al., 2021; Calabon et al., 2022; Xu et al., 2023). Among them, *Hermatomyces* and *Kirschsteiniothelia* have a worldwide distribution, while *Pleopunctum* are reported from China and Thailand (Liu et al., 2019; Phukhamsakda et al., 2020; Senwanna et al., 2021; Xu et al., 2023). To date, all published *Paramonodictys* species are described from China (Hyde et al., 2020; Yang et al., 2022; Xu et al., 2023). However, occurring at different altitudes suggests that *Paramonodictys* is highly adaptable to different environments and thus may also exist in other countries. *Sparticola* species mainly occur in Europe except for *S. muriformis* from China (Phukhamsakda et al., 2016; Karunarathna et al., 2017). In this study, we report *Sparticola* from freshwater habitat for the first time.

The classification of species in *Hermatomyces*, particularly *H. sphaericus*, has been widely debated. Koukol et al. (2018) considered *H. chromolaenae*, *H. saikhuensis* and *H. tectonae* as synonyms for *H. sphaericus*, a species that they regarded as monomorphic. Using the GCPSR method, Phukhamsakda et al. (2020) further supported this conclusion. Conversely, Tibpromma et al. (2018) identified *H. biconisporus* as a distinct species that produces two types of conidia, and clusters within *H. sphaericus* clade. They rejected Koukol’s treatment and suggested that *H. sphaericus* might be a species complex. The taxonomic statuses of two other species in the *H. sphaericus* clade, *H. biconisporus* and *H. pandanicola*, remain unresolved. However, Koukol and Delgado (2019) speculated that contamination during single spore isolation may have led to a mixture of conidia from *H. sphaericus* and *H. biconisporus*, while *H. pandanicola* might be a hybrid species, or the sequences in GenBank could have been provided erroneously (Koukol et al., 2018). Therefore, it is essential to collect fresh samples of *H. biconisporus* and *H. pandanicola* to resolve their taxonomic controversies.

## Data availability statement

The datasets presented in this study can be found in online repositories. The names of the repository/repositories and accession number(s) can be found in the article/supplementary material.

## Author contributions

JM provided the research materials. Y-ZZ and JM contributed to the methodology. N-GL performed the phylogenetic analyses. Y-ZZ, Q-LC, and N-GL wrote the original draft. Y-ZL, H-BC, and N-GL reviewed the draft. All authors contributed to manuscript revision, read, and approved the submitted version.

## Footnotes

^1^https://nmdc.cn/fungalnames/registe

^2^https://mafft.cbrc.jp/alignment/server/index.html

^3^http://tree.bio.ed.ac.uk/software/figtree
